# Detection of hemolytic Shiga toxin-producing *Escherichia coli* in fresh vegetables and efficiency of phytogenically synthesized silver nanoparticles by *Syzygium aromaticum* extract and gamma radiation against isolated pathogens

**DOI:** 10.1186/s12866-023-02994-8

**Published:** 2023-09-18

**Authors:** Hanan S. El-Bastawisy, Gharieb S. El-Sayyad, Feriala A. Abu Safe

**Affiliations:** 1 Drug Radiation Research Department, National Center for Radiation Research and Technology (NCRRT), Egyptian Atomic Energy Authority (EAEA), Cairo, Egypt; 2 Botany Department, Faculty of Women for Art, Science and Education, Ain Shams University, Cairo, Egypt

**Keywords:** Foodborne *E. coli*, Biogenic silver nanoparticles, Gamma irradiation

## Abstract

**Background:**

Shiga toxin-producing *E. coli* (STEC) is a major cause of foodborne diseases accompanied by several clinical illnesses in humans. This research aimed to isolate, identify, and combat STEC using novel alternative treatments, researchers have lately investigated using plant extract to produce nanoparticles in an environmentally acceptable way. At various gamma-ray doses, gamma irradiation is used to optimize the conditions for the biogenically synthesized silver nanoparticles (Ag NPs) using an aqueous extract of clove as a reducing and stabilizing agent.

**Methods:**

On a specific medium, 120 vegetable samples were screened to isolate STEC and molecularly identified using real-time PCR. Moreover, the antibacterial and antibiofilm activities of biogenically synthesized Ag NPs against the isolated STEC were examined.

**Results:**

Twenty-five out of 120 samples of eight types of fresh vegetables tested positive for *E. coli,* as confirmed by 16S rRNA, of which three were positive for the presence of Stx-coding genes, and six were partially hemolytic. Seven antibiotic disks were used to determine antibiotic susceptibility; the results indicated that isolate STX_2_EC had the highest antibiotic resistance. The results demonstrated that Ag NPs were highly effective against the STEC isolates, particularly the isolate with the highest drug resistance, with inhibition zones recorded as 19 mm for STX_2_EC, 11 mm for STX_1_EC_1_, and 10 mm for STX_1_EC_2_ at a concentration of 108 µg/mL. MICs of the isolates STX_1_EC_1_, and STX_1_EC_2_ were 13.5 µg/mL whereas it was detected as 6.75 µg/mL for STX_2_EC. The percentages of biofilm inhibition for STX_1_EC_2_, STX_1_EC_1_, and STX_2_EC, were 78.7%, 76.9%, and 71.19%, respectively.

**Conclusion:**

These findings suggest that the biogenic Ag NPs can be utilized as a new promising antibacterial agent to combat biofouling on surfaces.

**Supplementary Information:**

The online version contains supplementary material available at 10.1186/s12866-023-02994-8.

## Background

Foodborne organisms such as Shiga toxin-producing *E. coli* (STEC) can cause watery, bloody diarrhea, hemorrhagic colitis, and potentially fatal hemolytic uremic syndrome (HUS). Shiga toxins are divided into two subfamilies, *Stx1* and *Stx2*, and are encoded by *stx1* and *stx2* are the primary virulence factors of STEC [1]. They are pathogenic by adhering to and causing effacement lesions in the intestinal microvilli of the host cell. Strains of STEC are one of the most prevalent causes of foodborne illness worldwide [2]. STEC is transmitted through contaminated foods, contaminated water, and person-to-person transmission [3]. Fresh fruits and vegetables are excellent sources of nutrients, minerals, vitamins, and fibers and are essential for human health [4]. They are susceptible to microbial contamination in various ways, including contact with soil, dust, and water, as well as harvest and postharvest processing [5]. The use of untreated wastewater and manure as fertilizer in the cultivation of fruits and vegetables is a significant source of pollution [6]. Contact with animal manure, sewage, or irrigation water, as well as transport and merchant handling, can all result in contamination [7]. In recent years, human illness outbreaks linked to the consumption of fresh or minimally processed fruits and vegetables have increased [8].

Nanotechnology is the manipulation of nanomaterials for various applications, including its vital role in the food and agriculture sectors, contributing to crop enhancement, improving food quality and safety, and promoting human health through novel approaches [9].

As antimicrobial compounds, metals are regarded as a promising method for inhibiting microbial growth. Heavy metals were assumed to have broad-spectrum biocidal effects. It is believed that ionic silver has the highest antibacterial activity of any metal, along with long-lasting biocidal properties and low toxicity to eukaryotic cells [10].

Gamma (γ) irradiation is a powerful technology that should be considered for eco-friendly nanoparticle synthesis [11]. Since gamma irradiation does not require a chemical initiator for reduction, irradiation is known as green technology [12, 13]. It is a straightforward, hygienic process that uses an aqueous solution (as a water source) at room temperature and pressure. Gamma-irradiation is controlled by irradiation dose, and the NPs produced are extremely pure, fully reduced, and chemical-free [14].

Employing biological systems to produce metallic nanoparticles has numerous benefits. Green strategies reduce the use of expensive chemicals, require less energy, and generate eco-friendly products and byproducts. According to the published references [15, 16], and literature point of view, the biological synthesis possessed some advantages including being more economically viable and less hazardous than the chemical and physical methods.

According to numerous publications, Ag NPs were synthesized using a variety of plants by using plant extract as a bio-reducing agent and metallic salt as a precursor ([17, 18], and [19]).

Clove (*Syzygium aromaticum*) is a widely used spice, as a food preservative and medicinal herb. Cloves contain various active compounds, such as eugenol, eugenone, chavicol, methyl salicylate, and caryophyllene; and flavonoids, such as eugenin, kaempferol, and eugenitin; triterpenoids such as oleanolic acid, and numerous sesquiterpenes with antimicrobial and anticancer activities [20].

The objective and motivation of this study were to determine the microbiological quality and safety of several fresh vegetables, as well as the green synthesis of Ag NPs using the natural precursor of *Syzygium aromaticum* and gamma radiation in order to assess their antibacterial and antibiofilm properties against Shiga toxin-producing *E. coli.*

## Materials and methods

### Chemicals and reagents

For the synthesis of NPs, analytical-grade chemicals including silver nitrate (Sigma Aldrich, UK) were used. However, media for microbiological assay (Eosin Methylene Blue (EMB), and MacConkey) were purchased from Oxoid in the UK.

## Collection of samples

A total of 120 samples of eight types of fresh vegetables, including Cucumber *(Cucumis sativus*), Tomato (*Solanum lycopersicum*), Carrot (*Daucus carota*), Green pepper (*Capsicum annuum*), Lettuce (*Lactuca sativa*), Parsley (*Petroselinum crispum*), dill (*Anethum graveolens*), and Coriander (*Coriandrum sativum*) approximately 15 samples of each type were collected from local markets, Cairo, Egypt. The samples were transported to the laboratory within 24 h in insulated bags and kept at 4 °C until microbial analysis, either immediately upon arrival at the laboratory or after being refrigerated overnight storage at 4 °C [21].

## Bacteriological analysis

### A. Isolation of Shiga toxin-producing *Escherichia coli* (STEC)

Each sample was washed with sterile, distilled water. Each vegetable sample (25 g) was homogenized in a sterile blender, suspended in 225 mL of buffered peptone water (Oxoid) as an enrichment medium, and incubated at 37 °C for 18–24 h according to the method described by the following references [22] & [23].

One hundred µL of the enriched culture was streaked on eosin methylene blue (EMB) agar for *E. coli* isolation (selective and differential medium). On EMB, colonies with a greenish metallic sheen and black cores were categorized as *E. coli* (Figure S1). To differentiate non-pathogenic from enterohaemorrhagic *E. coli*, these colonies were isolated, streaked on MacConkey agar with Sorbitol (SMAC), and incubated for 24 h at 37 °C [24]; [25]. Using 16S rRNA-PCR, colorless colonies were isolated and identified as *E. coli*. Blood hemolysis was evaluated for *E. coli* isolates.

### B. Detection of bacterial counts of the tested vegetables

Each sample was homogenized and suspended in flasks containing 225 mL of sterile saline (0.9%), shaken for 2 h, serially diluted up to 10^−7^, and spread-plated in duplicate on pre-solidified nutrient agar plates (Oxoid). Inoculated plates were incubated at 37 °C for 24–48 h. Plates with 30 to 300 colonies were considered [26].

### Molecular identification of prospective *E. coli* isolates and Shiga toxin-producing *E. coli*

DNA extraction from samples was carried out using the QIAamp DNA Mini kit (Qiagen, Germany, GmbH) according to the manufacturer’s recommendations. Primers used were supplied from Metabion (Germany) and are listed in Table 1. The products of PCR were separated by electrophoresis on 1.5 % agarose gel (Applichem, Germany, GmbH. The gel was photographed by a gel documentation system (Alpha Innotech, Biometra) and the data was analyzed through computer software.

**Table 1:** Primers sequences, target genes, amplicon sizes and cycling conditions

Target gene	Primers sequences	Amplified segment (bp)	Primary denaturation	Amplification (35 cycles)			Final extension	Reference
				Secondary denaturation	Annealing	Extension		
*E. coli phoA*	CGATTCTGGAAATGGCAAAAG	720	94˚C 5 min	94˚C 30 s	55˚C 40 s	72˚C 45 s	72˚C 10 min	[27]
	CGTGATCAGCGGTGACTATGAC							
*Stx1*	ACACTGGATGATCTCAGTGG	614	94˚C 5 min	94˚C 30 s	58˚C 40 s	72˚C 45 s	72˚C 10 min	[28]
	CTGAATCCCCCTCCATTATG							
*Stx2*	CCATGACAACGGACAGCAGTT	779	94˚C 5 min	94˚C 30 s	58˚C 40 s	72˚C 45 s	72˚C 10 min	
	CCTGTCAACTGAGCAGCACTTTG							

### Preparation of cloves (*Syzygium aromaticum*) extract

**Fig. 1:**
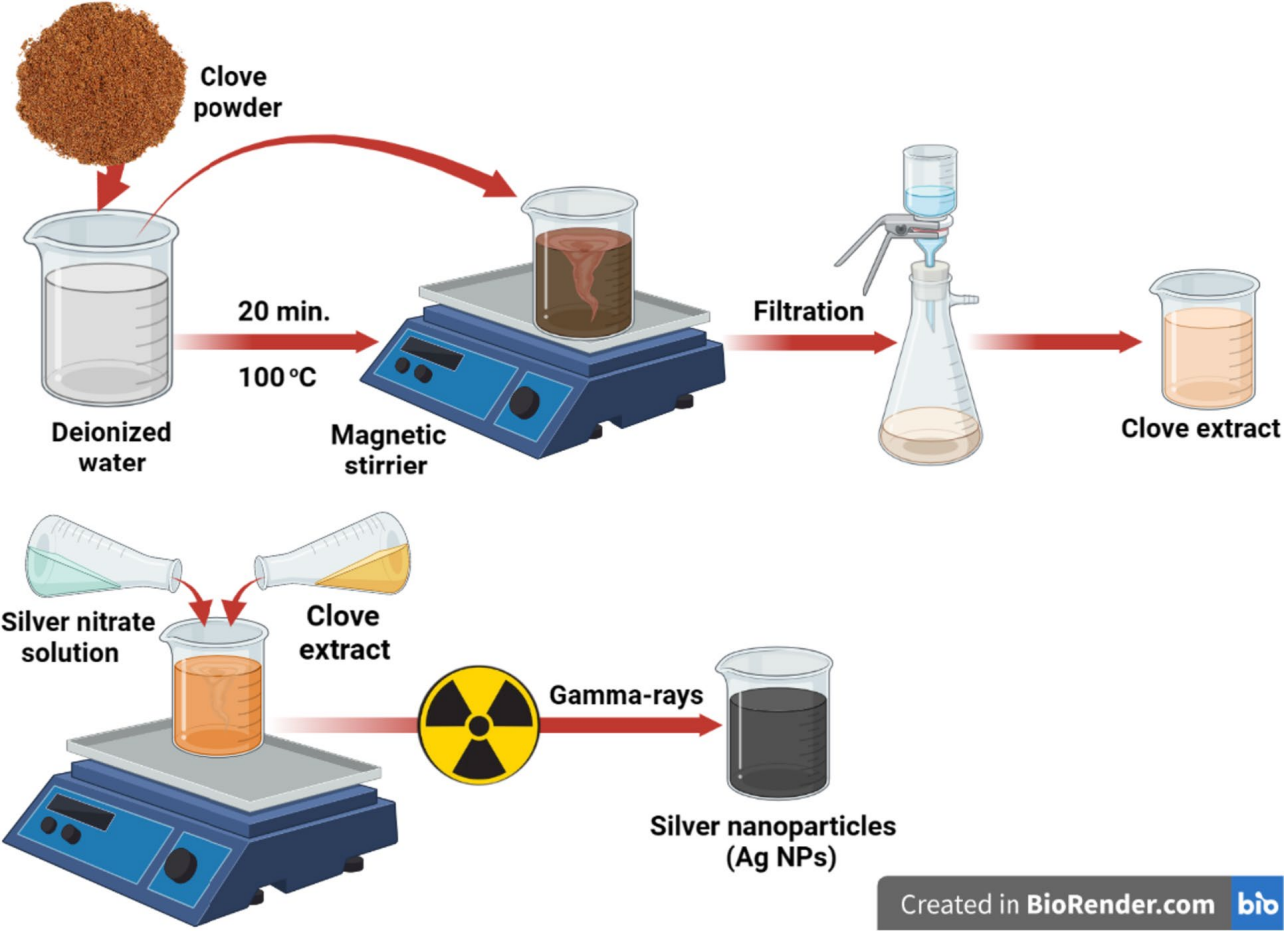
The synthetic steps for the preparation of clove aqueous extract and Ag NPs biosynthesis by the clove aqueous extract in the presence of gamma rays. The figure created by BioRender.com

Clove aqueous extract was produced by bringing 0.5 g of powdered clove buds and 400 mL of distilled water to a boil for 20 min. The resulting extract was filtered through Whatman No. 1 filter paper and stored at 4 °C for further experiments as shown in Fig. 1 [19].

### Biosynthesis of silver nanoparticles (Ag NPs) using extract of clove buds and gamma irradiation

The green synthesis of Ag NPs was achieved by mixing eight milliliters of clove extract with 40 mL of 2 mM Ag NO_3_. The ratio between Ag NO_3_ and the clove extract was chosen according to the recently published paper [19]. This mixture was maintained in the dark for 24 h to prevent the photoactivation of Ag NO_3_ and at room temperature as a control [19]. Another screening experiment was conducted to determine the effect of gamma radiation at doses of 3, 5, 10, and 20 kGy on the reduction of silver ions. The change in color of the solution from yellow to dark brown indicated the successful production of Ag NPs as shown in Fig. 1.

### Gamma irradiation

Gamma irradiation was carried out at National Center for Radiation Research and Technology (NCRRT), Cairo, Egypt. The source of the radiation was a ^60^Co-Gamma chamber 4000-A-India. At the time of the experiment, the dose rate was 1.10 kGy/h. Gamma irradiation generates solvated electrons, hydrated radicals, and free radicals through water radiolysis.

### Characterization of the silver nanoparticles

A UV–Vis. spectrophotometer was used to analyze the optical properties of Ag NPs (JASCOV-560. UV–Vis. spectrophotometer). Moreover, X-ray diffraction was used to determine the crystallinity, crystallite size, and/or lattice of the produced Ag NPs were determined (the XRD-6000 lists, Shimadzu apparatus, SSI, Japan. Diffracted angle 2θ was used to measure the intensity of the diffracted X-rays. The size and distribution of the most prevalent Ag NPs (DLS-PSS-NICOMP 380-USA) were determined using dynamic light scattering. Additionally, a high-resolution transmission electron microscope was utilized to examine the microstructure, mean particle size, and shape of the synthesized Ag NPs (HRTEM, JEM2100, Jeol, Japan). Scanning electron microscopy (SEM) ZEISS EVO15-UK was used to examine the surface morphology of Ag NPs. The elemental structure, purity, and proportion of each metal contained in our samples were estimated using Energy Dispersive X-ray (EDX) analysis. The mapping technique was used following SEM/EDX analysis to get comprehensive information on the purity, distribution, and location of the metals detected in the synthesized Ag NPs. Lastly, Fourier transform infrared spectrum analysis (FT–IR) was a crucial objective that revealed the chemical functional groups present in both aqueous clove extract and clove extract-Ag NPs. Using a JASCO FT–IR 3600 infrared spectrometer, samples were mixed with KBr and pressed before being analyzed. It was measured at a wavenumber range from 400 to 4000 cm^−1^.

## Antibacterial activity assays

### A. Antibiotic susceptibility test

*E. coli* isolates were tested for antibiotic sensitivity using the disk diffusion agar method [29], as recommended by the Clinical and Laboratory Standard Institute [30]. The following conventional antibiotic discs (μg) (Oxoid) were used; amoxicillin (AML 10), amoxicillin/ clavulanic acid (AMC 30), ampicillin (AMP 10), ampicillin-sulbactam (SAM 20), chloramphenicol (C 30), gentamicin (CN 10) and penicillin G (P 10).

### B. Antibacterial activity of Ag NPs against STEC bacteria

The antibacterial properties of Ag NPs, Ag NO_3_, and clove extract against STEC were investigated using the agar well diffusion technique. A bacterial suspension was adjusted to a standard concentration of 0.5 McFarland, equal to (1 × 10^8^ CFU/mL). Bacteria were inoculated on nutrient agar medium, and 6.0 mm wells containing 100 µL of the tested treatments were drilled into the top of each nutrient agar plate. A disk of gentamicin was used as a positive control. All experiments were conducted in triplicate, and the plates were incubated at 37 °C for 24 h. The microbial growth inhibition zone was measured in mm [31].

Thus, the minimum inhibitory concentration (MIC) was determined using the microdilution assay [32]. Briefly, STEC was grown on nutrient agar, and three or four colonies were suspended in fresh sterile nutrient broth to achieve a concentration of 1 × 10^8^ CFU/mL. Each well of a 96-well microtiter plate was filled with one hundred microliters of the 1:100 diluted cell suspensions. The STEC was exposed to a twofold dilution series of Ag NPs (starting with 108 μg/mL concentration till 1.687 μg/mL). The specific and fixed wavelength used for detecting the optical density for the bacterial growth (at 600 nm) which totally different from the optical density for the synthesized Ag NPs. A negative control (the nutrient broth alone) and positive control (the tested microbes and the nutrient broth) were conducted for these purposes, additionally, another control like the synthesized Ag NPs alone was used to compare the conducted results as an auto-zero purpose. The MICs were determined as the lowest nanoparticle dilution capable of inhibiting visible bacterial growth.

### The efficiency of Ag NPs as biofilm inhibitors

Protocol for biofilm detection was carried out according to the reference [33]. Briefly, various STEC isolates were grown in tryptic soy broth containing various concentrations of Ag NPs (0, 6.75, and 13.5 µg/mL) using microtiter 96-well plates and incubated at 37 °C for 24 h.

The wells were rinsed twice with phosphate buffered saline (PBS) and once with double-distilled water following the incubation period. The biofilm was stained for 10 min with 100 µL of 0.1% crystal violet and washed once with double-distilled water. Finally, crystal violet was solubilized with 33% acetic acid, and the optical density at 595 nm was measured (Microplate Reader-HUMA HS) to determine biofilm formation.

### Scanning electron microscopy of the control and treated STEC cells

The bacterial cells collected from the antibiofilm test were rinsed with PBS and stored in 5.0 mL glutaraldehyde (3.5%). The maintained bacterial cells were repeatedly washed with PBS before dehydration by washing them in a series of ethanol solutions with concentrations of 30, 50, 70, 80, 95, and 100 % for 10 min, which was an essential step in the drying process [34]. For the imaging technique, the bacterial cells were adhered to the metal stubs and placed above them. Scanning electron microscopy (SEM-ZEISS, EVO-15-UK) was used to investigate the surface morphology of non-treated and treated bacterial cells using the biosynthesized Ag NPs.

### Statistical analysis

The statistical analysis was conducted using GraphPad Prism version 5 for Windows 10 (GraphPad Software®, San Diego, CA, USA; www.graphpad.com). One-way analysis of variance with the Tukey multiple comparisons test, was used. The mean ± standard deviation of data from three replications was reported (SD). A *P*-value of 0.05 indicated statistical significance.

## Results

### Microbiological analysis of fresh vegetables and total bacterial count

The prevalence of *E. coli* found in the different tested vegetables was shown in Table 2. Our results indicate that out of 120 vegetable samples tested for *E. coli* contamination (isolated on EMB agar plate and confirmed using 16SrRNA-PCR), 20.8% (25 instances) were identified. *E. coli* was not detected in parsley (*Petroselinum crispum*) and dill (*Anethum graveolens*) samples. In contrast, the results indicated that 9 (60%) Cucumber (*Cucumis sativus*) samples were positive for *E. coli*, followed by Tomato (*Solanum lycopersicum*) with 7 (46%), Carrot (*Daucus carota*) with 4 (26%), Green pepper (*Capsicum annuum*) with 3 (20%), and Lettuce (*Lactuca sativa*) and Coriander (*Coriandrum sativum*) with 1 (7%). According to the results, only six *E. coli* isolates were validated as partial blood hemolytic (data not shown). In the current study, 16SrRNA-PCR revealed that three out of 25 *E. coli* isolates (12%) were STEC isolates (two isolates have *Stx1* and one has *Stx2*).

**Table 2:** The total bacterial count and prevalence of *E. coli* of the tested fresh vegetables

Sample	No. of samples	Prevalence of *E. coli*		Total bacterial count average (log CFU/mL)
		**No.**	**%**	
1- Cucumber *(Cucumis sativus*)	15	9	60	6.6
2- Tomato (*Solanum lycopersicum*)	15	7	46	5.7
3- Carrot (*Daucus carota*)	15	4	26	6.8
4- Green pepper (*Capsicum annuum*)	15	3	20	5.8
5- Coriander (*Coriandrum sativum*)	15	1	7	6.3
6- Lettuce (*Lactuca sativa*)	15	1	7	6.9
7- dill (*Anethum graveolens*)	15	-	-	5.5
8- Parsley (Petroselinum crispum)	15	-	-	5.8
**Total number of samples**	**120**	**25**	**20.8**	

For total bacterial count, the average bacterial count ranged between 5.5 and 6.9 log CFU/g based on the microbiological analysis of the aforementioned eight types of fresh vegetable samples. The highest concentration was detected in lettuce (6.9 log CFU/mL), followed by carrot (6.8 log CFU/mL), cucumber (6.6 log CFU/mL), and coriander (6.3 log CFU/mL). It was noticed that the total bacterial count of parsley and green pepper samples was 5.8 log CFU/mL, whereas it was (5.7 log CFU/mL) for tomatoes. On the other hand, comparatively, a lower concentration was recorded in dill with a count of 5.5 log CFU/mL. Table 2 depicted the microbiological analysis of the above eight types of fresh vegetables.

### Molecular identification of prospective *E. coli* isolates and STEC

**Fig. 2:**
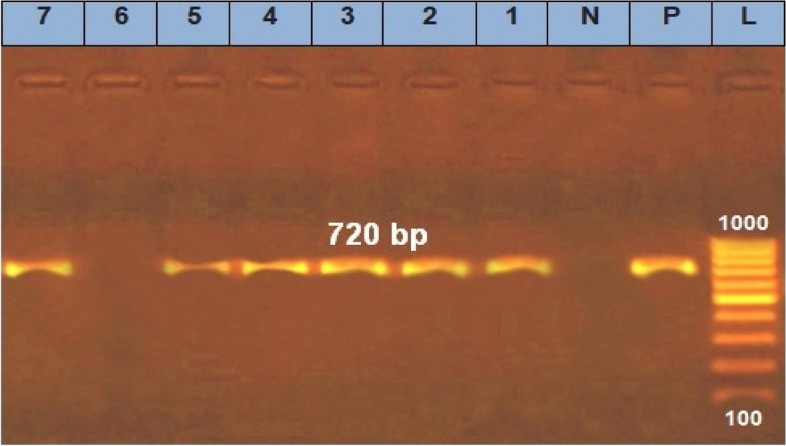
Agarose gel electrophoresis of PCR of specific primer (phoA) for characterization of Enterohemorrhagic *E*. *coli* from vegetables samples. Photograph (1) showed that lane (L) represent 100 bp ladder as molecular size DNA marker, whereas lane (P) is the positive control for phoA gene of E.coli and lane (N) is the negative control. Lanes of our tested isolates were 1, 2, 3, 4, 5 and 7 are positive *E*. *coli* strains and their bands appear at 720 bp which is typical the positive control but lane 6 is negative

**Fig. 3:**
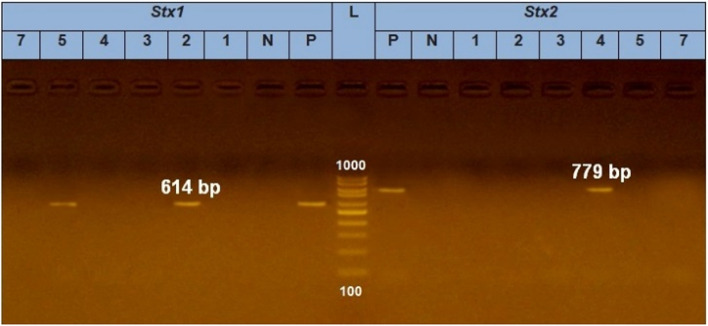
Agarose gel electrophoresis for detection of Shiga toxin-producing *Escherichia* *coli*. Photograph (2) showed that lane (L) represents 100 bp ladder as molecular size DNA marker, whereas lane (P) on the left side is the positive control for Stx1 gene but on the right side is the positive control of Stx2 gene. Lane (N) is the negative control of each. Lanes 2 and 5 on the left side are positive for Stx1 with bands appear at 614 bp while lane number 4 on the right side is positive for Stx2 with a band appear at 779bp

In this study, a real-time PCR assay was used to confirm the diagnosis of the *E. coli* by using *phoA, Stx1, Stx2* genes and the result of the PCR assay and bacterial culture are identical, indicating that PCR assay was more sensitive for detecting this organism. We examined three genes: *phoA*, *Stx1* and *Stx2,* PCR results showed that the gene *phoA* was detected in six of the selected seven strains, while *Stx1* and *Stx2* genes of virulent *E. coli* were positive in three of the strains Figs. 2 and 3.

### Characterization of the synthesized silver nanoparticles

#### UV–Vis. spectroscopy analysis

**Fig. 4:**
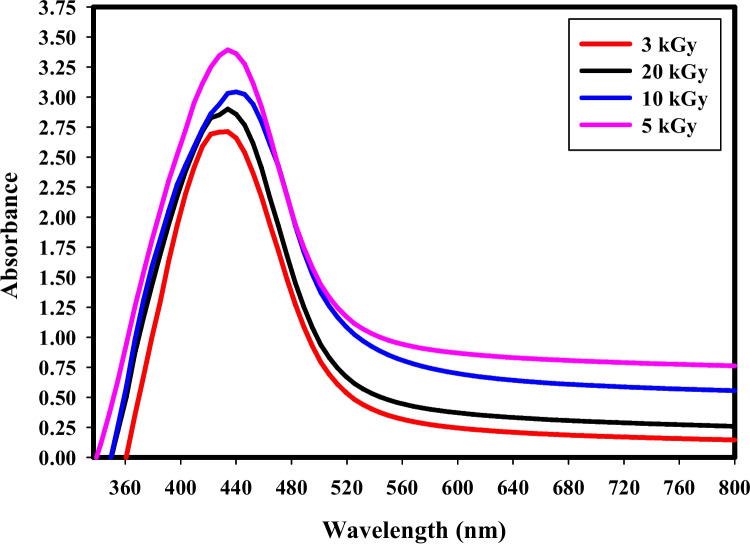
The UV–Visible absorption spectrum of biosynthesized Ag NPs at different gamma rays doses

UV–Vis. spectroscopy was carried out to validate the synthesis of Ag NPs. Figure 4 demonstrates that the absorption peak was established between 435 and 450 nm. The color of the mixture of Ag NO_3_ and clove aqueous extract was pale yellow, but it darkened forming dark brown color when Ag NPs were produced. The absence of the UV absorption band at 280 nm, which corresponds to clusters of Ag^+^ ions in the solution, is depicted in Fig. 4. It was discovered that increasing the gamma irradiation dose beyond 5.0 kGy increased the size distribution and mean particle size of the produced Ag NPs. The results demonstrated that the product amount and size of the generated Ag NPs are adequately regulated by gamma irradiation. The amount of Ag NPs is greatest with a gamma dosage of 5 kGy with an absorption band at 430 nm.

### XRD analysis

**Fig. 5:**
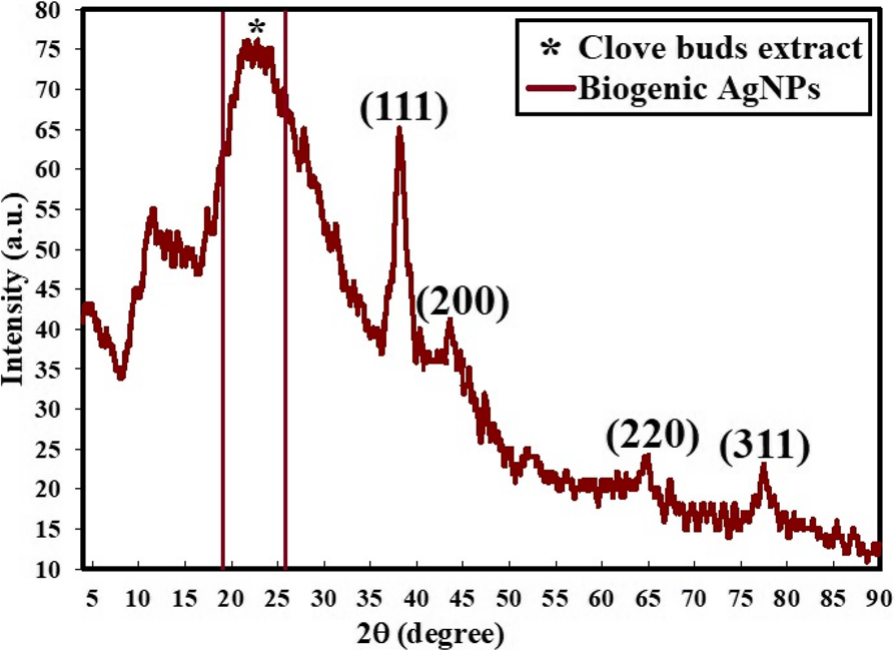
X-ray diffraction showing crystalline Ag NPs synthesized from aqueous extract of clove and gamma irradiation at 5 kGy

From XRD it was evaluated that Ag NPs exhibits crystalline nature with prominent peak as shown in Fig. 5. The crystal size corresponding to intense peak was determined using Scherrer’s relation and found that the average crystal size is 7.6 nm.

1 $$\mathrm{D}=\mathrm{K\lambda }/\mathrm{\beta cos \theta }$$

Here k is Scherrer constant having value 0.94, λ is the wavelength of X-ray, β is full width at half maxima (FWHM) which is calculated by fitting a single Gaussian curve, θ is Bragg diffraction angle and D is the particle size. Figure 5 illustrates a typical XRD spectrum of silver nanoparticles prepared by the green method. The XRD peak at 2θ = 38.17° could be attributed to the (111) crystallographic plane. Figure 5 presents XRD diffraction peaks of Ag NPs including peaks at 2θ = 38.17°, 44.19°, 64.98°, and 78.31° which matched with a standard card JCPDS-ICDD card 04–0783, and corresponding to (111), (200), (220), and (311) Bragg’s reflections, respectively. It must be noted that, the distinct peak at 2θ = 22.15° could be recognized to the amorphous nature of clove extract.

### High-resolution transmission electron microscopy (HRTEM), scanning electron microscope (SEM) and dynamic light scattering (DLS) examinations

**Fig. 6:**
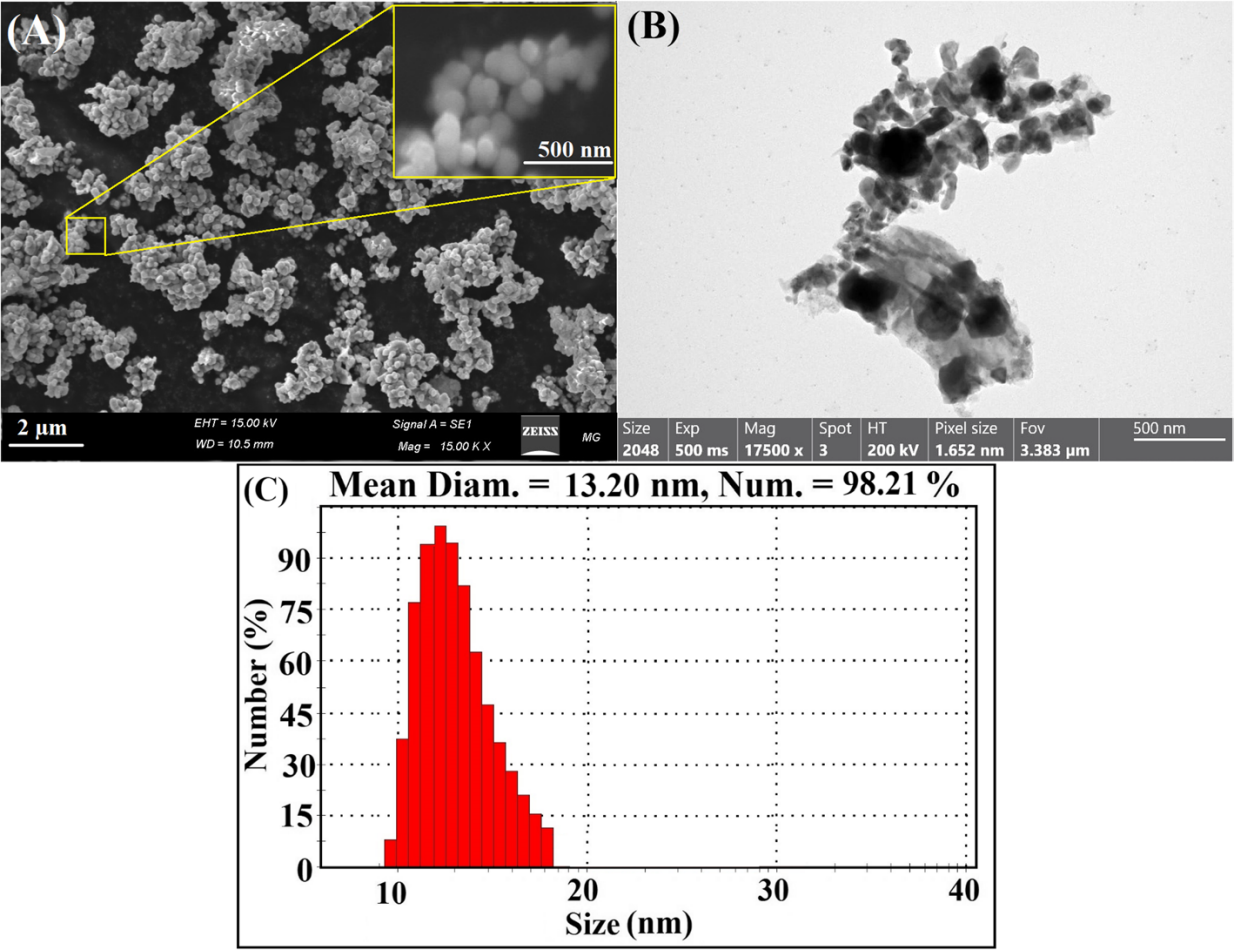
**A** SEM of spherical Ag NPs, **B** Shape, microstructure (HRTEM) of Ag NPs synthesized by clove aqueous extract and gamma irradiation at 5 kGy., and **C**-he mean diameter of Ag NPs (13.2 nm) using DLS technique

SEM and HRTEM were used to investigate the average particle size and the characteristic shape of the synthesized Ag NPs, and the results were correlated to the DLS study, which was used to determine the mean and most prevalent particle size distribution. As depicted in Fig. 6A and B, the spherical shapes with mono-dispersed NPs of the biogenic Ag NPs with an average size ranging from 9.18 to 12.98 nm and a mean diameter of 10.99 nm were visible in the SEM and HRTEM images of (Fig. 6A and B). The DLS method was also used to estimate the average particle size. As shown in Fig. 6, the average particle size of Ag NPs generated by clove aqueous extract and 5 kGy of gamma irradiation was determined to be 13.2 nm (Fig. 6C). It was observed that the particle size distribution measured using the DLS technique was greater than the average particle size determined using the HRTEM image.

### Energy dispersive spectroscopy (EDX) and mapping analysis

**Fig. 7:**
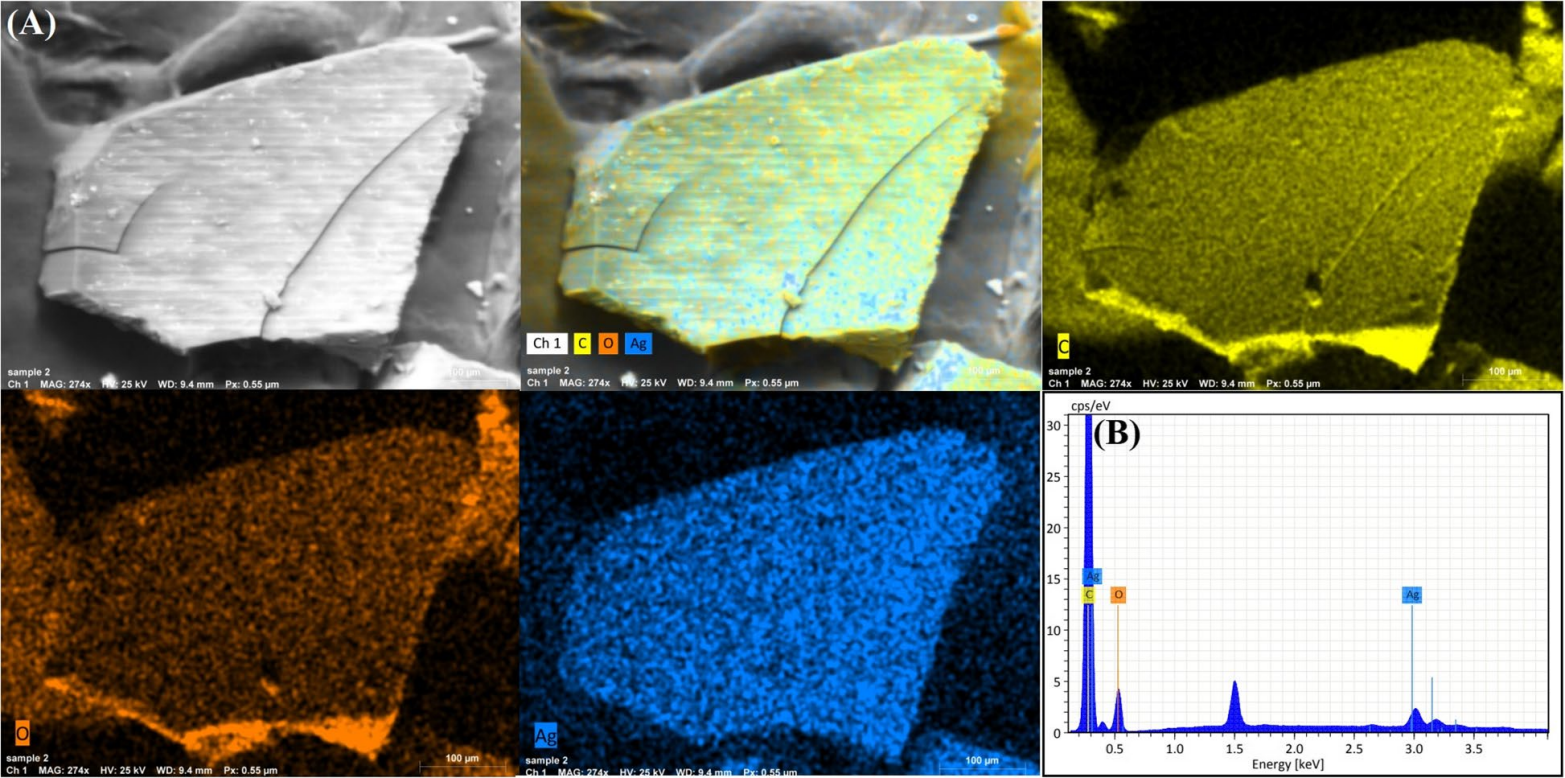
**A** SEM/EDX mapping images, and **B** EDX analysis of the biogenically synthesized Ag NPs using clove aqueous extract and gamma irradiation at 5 kGy

Figure 7A shows the elemental mapping images of Ag NPs-clove extract. The models are designated as Ag, C, and O based on their distribution. In addition, the clove extract was represented by the atoms C and O. As depicted in the figure, the Ag NPs (bright NPs; blue) was then uniformly dispersed over the clove extract atoms (C, and O). EDX is utilized for the elemental analysis of biosynthesized nanoparticles. The EDX profile depicted in Fig. 7B reveals a strong signal for the Ag atoms present, in addition to C and O biomolecules (clove extract biomolecules) participating in the capping of the surface of these nanoparticles. The 3 keV absorption peak is typical of silver nanocrystals

### Fourier transform infrared spectroscopy (FTIR) analysis

Using FT–IR (Perkin Elmer Spectrum, Jasco-6100) in the 4000 to 400 cm^−1^ wavelength range, the biomolecules relevant to the production of silver nanoparticles mediated by clove bud extract and responsible for the conversion of Ag^+^ to Ag^o^ were identified. (Figure S2). The spectra of clove extract display a prominent peak at 3319.2 cm^−1^ that was shifted to 3263.61 cm^−1^ for -OH groups owing to silver nanoparticle interaction. The peak at 2016 cm^−1^ might be attributed to the C-H stretching of the alkane group. The bands in the 1700–1600 cm^−1^ range represent C = O stretching vibrations. These findings revealed the existence of numerous biomolecules that may serve as reducing and stabilizing agents during the synthesis of clove extract-Ag NPs.

### Antibiotic resistance of the STEC isolates

**Fig. 8:**
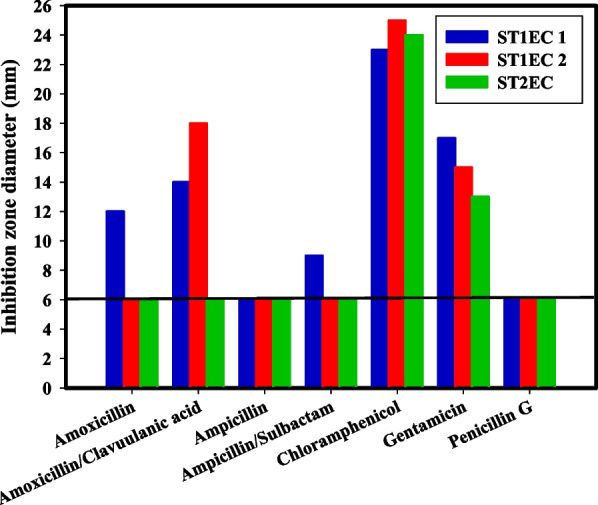
The susceptibility test of the 3 identified Shiga toxin-producing *E*. *coli*, 6 mm indicated negative result

Antibiotic treatment of STEC infections is generally not recommended due to the risk of progression to HUS. Consequently, new anti-infection strategies must be developed. The mean inhibition zones for each isolate of STEC (STX_1_EC_1_, STX_1_EC_2_, and STX_2_EC) were measured with seven antibiotics. The results indicated that isolate STX_2_EC was sensitive to two antibiotics, chloramphenicol and gentamicin; isolate STX_1_EC_2_ was sensitive to three antibiotics, chloramphenicol, gentamicin, and amoxicillin/clavulanic acid; and isolate STX_1_EC_1_ was sensitive to five of seven antibiotics. The antibiotic sensitivity test results indicate that the STX_2_EC isolate has the highest level of antibiotic resistance, followed by STX_1_EC_2_ and STX_1_EC_1_ (Fig. 8).

### Antibacterial activity of Ag NPs against STEC isolates

**Fig. 9:**
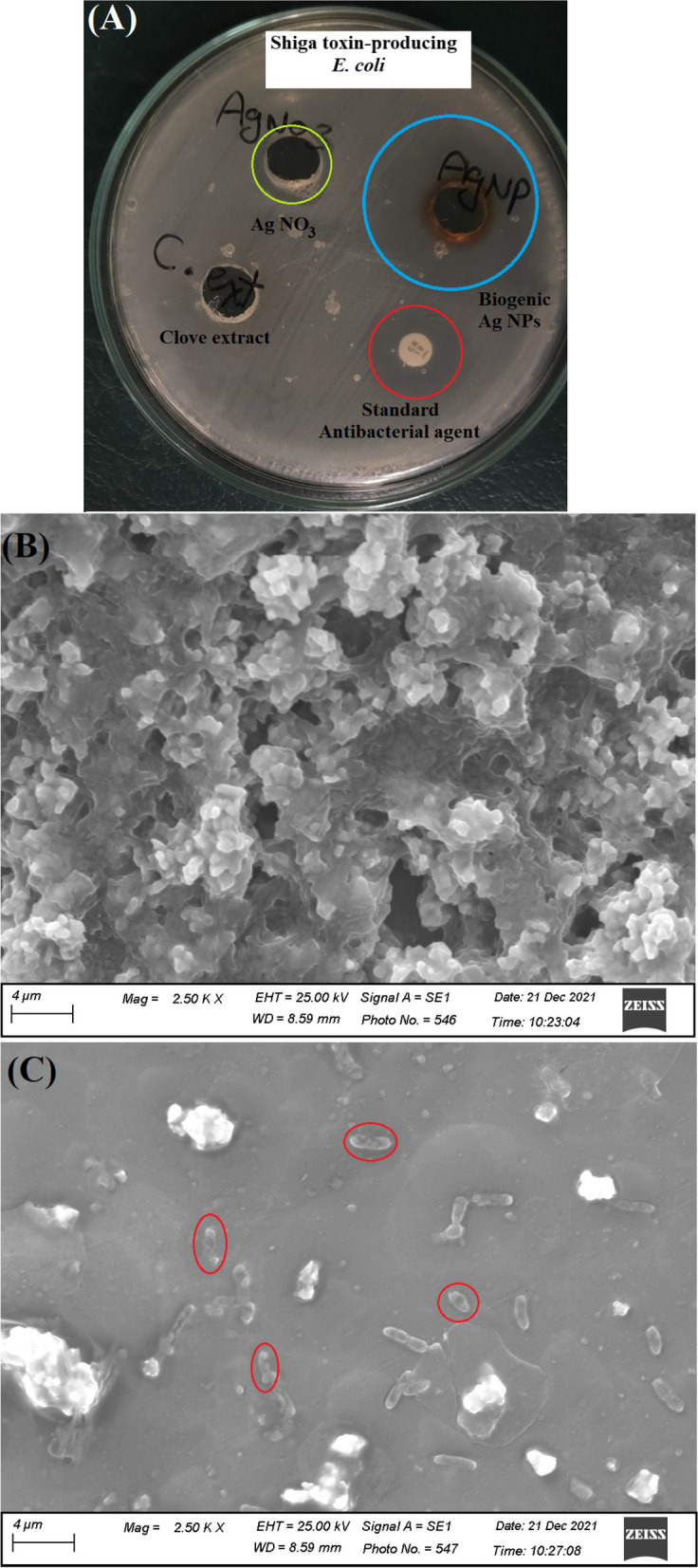
The efficiency of Ag NPs as anti-STEC and anti-biofilm activities where, **A** ZOI Antimicrobial activity of the biogenic Ag NPs, Ag NO_3_, clove aqueous extract and conventional antibiotic discs (CN 10), **B** highly biofilm producing bacterial cells without Ag NPs treatment, and **C** the decreased and deformed bacterial cells under the influence of Ag NPs

In the present study it was noticed that Ag NPs were highly effective against the STEC isolates (as shown in Fig. 9) specially the highly drug resistance isolate STX_2_EC (19 ± 2.3 mm) inhibition zone and (11 ± 1.73 and 10 ± 1.73 mm) inhibition zone for STX_1_EC_1_ and STX_1_EC_2_, respectively; values are represented as mean ± SD. Whereas the MIC for ST_2_EC was 6.75 µg/mL and (13.5 µg/mL) for STX_1_EC_1_ and STX_1_EC_2_ of Ag NPs.

Figure 9B indicated that normal *E. coli* cells appeared embedded in abundant biofilm secretions; however, after treatment with Ag NPs (Fig. 9C), the bacterial cells were deformed, with a noticeable decrease in the viable count, and the heavy biofilm was hindered. The treated bacterial cells were drastically altered and displayed severe damage, as seen by the creation of pits in their cell walls (indicated by the red circles)

### Efficiency of Ag NPs as biofilm inhibitors

Biofilm formation is frequent in pathogenic bacteria that release exopolysaccharide compounds. The anti-biofilm activity of Ag NPs against STEC is critical since biofilm development is essential for STEC colonization in reservoir animals as well as in green leafy crops. The maximum inhibition percentage was found to be 78.7% for STX_1_EC_2_, 76.9% for STX_1_EC_1_ at 6.75 µg/mL, and 71.19% for STX_2_EC generating strain at 3.375 µg/mL.

**Fig. 10:**
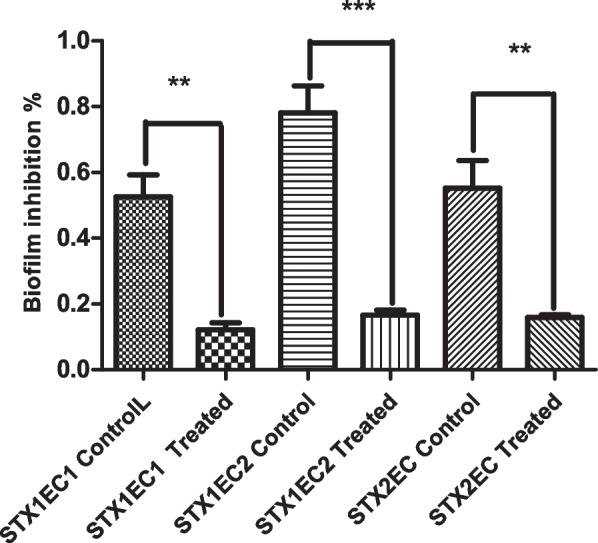
Efficiency of Ag NPs as biofilm inhibitors; the data are represented in the form of a bar graph and plotted using mean ± SD of three replicates (** *p* value: < 0.01 and *** *p* value: < 0.001)

The development of innovative Ag NPs-based anti-biofilm agents against STEC might aid in the design of ways to combat STEC infection and transmission. Overall, the current study shows that Ag NPs have strong anti-biofilm action against STEC isolates (as shown in Fig. 10). More research should be conducted to determine the mode of action of Ag NPs in order to disclose antibacterial and anti-biofilm capabilities.

## Discussion

The prevalence of *E. coli* in the tested vegetables samples may be attributed to that* E. coli* is a part of the normal microflora of the human and animal digestive tracts that has a biphasic existence and may efficiently live either in the host or in the environment where it is discharged with human sewage and animal excreta. Solomon et al. [3], cleared that, *Escherichia coli* O157:H7 is increasingly being isolated from fresh food, including bean sprouts, cantaloupes, apples, and leaf lettuce. The processes by which the pathogen enters the plant are unknown; however, one theory is that the plant becomes infected when cultivated in areas fertilized with inadequately treated manure. According to epidemiological data, *E. coli* O157:H7 can be found in up to 8.3% of dairy and beef cattle and is shed asymptomatically in the feces. Current manure-handling rules recommend composting manure before applying it to a field as fertilizer.

Previous studies carried out in different countries showed heterogeneous *E. coli* prevalence data in vegetables, in Brazil, [35] detected *E. coli* in 53% of the leafy vegetable samples; in Istanbul, Turkey, [36] showed that 60% of vegetable samples were positive for *E. coli*; in Iran, around 50% vegetables were contaminated with *E. coli* and from these, 87% were verotoxin *E.coli* [37]. On the contrary, in some other countries, the lower prevalence was reported. In Finland, *E. coli* was isolated from 15% of packaged fresh leafy vegetables [38]. A similar percentage was detected in lettuce from Canada [39] and in Switzerland, it was only found in 2% of lettuce [40].

According to the microbiological analysis, the tested samples were considered acceptable food according to the suggested microbiological recommendation by [41]: aerobic plate count (APC) < 10^7^ CFU/g was a reliable food. Also, [42] showed that raw, ready-to-eat vegetables are considered good and acceptable food when their microbial load lies beneath 7 log CFU/g since APC reflects the quality of vegetables because mesophilic bacteria are often present at levels up to 10^7^ CFU/g. The PCR assay and *E. coli* culture yielded identical results, demonstrating that the PCR method was more sensitive for identifying this pathogen. This result is consistent with [43] who recorded an accuracy of real-time PCR for detection of *E. coli* O111 and O157 and they concluded that this assay was quick diagnostic method for the presence or absence of *E. coli* strains. The molecular results for *E. coli* PCR using sets of primers were used for genotypic detection of virulence genes that may play a role in pathogenicity of *E. coli*. [44] cleared that *stx1* and *stx2* genes cause hemorrhagic uremic syndrome (HUS) which poses a great danger to the health of humans owing to its high virulence potential, low infectious dose (10–100 CFUs), persistence in the environment, and resistance to treatment options.

The synthesized Ag NP solutions exhibited an intense dark brown color due to the excitation of surface Plasmon oscillations in biogenic Ag NPs and served as a spectroscopic indicator of their production [19], with absorption peak was established between 435 and 450 nm, which was confirmed to be unique to Ag NPs [45]. The absence of the absorption UV band located at (280 nm) indicated that all silver ions are converted to Ag NPs in the first stage of the gamma irradiation-induced reduction reaction of silver ions by different doses of gamma radiation from 3 up to 20 kGy [46]. For the migration and charge transfer of Ag NPs to form Ag ions, it must be noted that, the clove aqueous extract used in our paper serve as a biological capping agent which prevent Ag ions formation and the change occurs at the acidic pH which not available in our synthetic process. Increasing the size distribution and mean particle size of the produced Ag NPs with increasing the gamma irradiation dose beyond 5.0 kG may be owing to the aggregation of the synthesized Ag NPs with the aid of additional electrons and free radicals formed during the reaction of gamma radiolysis of water [47]. The absorption bands change to a longer wavelength range of 430 nm to 450 nm, matching the dipole resonance of Ag NPs and indicating the homogeneity of spherical morphology with an indication of particle size increase [48].

From the XRD pattern, it was clear that the synthesized nanoparticles were made of metallic silver and they were predominantly face centered cubic (FCC) crystalline [49]. It was noticed that the average particle size investigated by DLS is greater than that detected by HRTEM; this may be because the DLS method estimated the hydrodynamic radius found around the synthesized Ag NPs surrounded by the water particles in relation to the large sizes of the capped Ag NPs [34]. The EDX profile reveals a 3 keV absorption peak similar to that detected by [50] which is characteristic of silver nanocrystals. The FTIR spectrum revealed that several functional groups, such as carboxylic and hydroxyl, were bound to silver, potentially stabilizing the nanoparticles and preventing their aggregation by proteins or polysaccharides [51, 52]. The current antibiotic resistance was similar to a previous study by [33].Our results are in accordance with [53] who showed that antibiotic usage may enhance antibiotic resistance among some foodborne infections. Antibiotic resistance in STEC, particularly to promising antibiotics such as azithromycin, may render these treatment choices ineffective in controlling STEC during the early phases of human infection.

For certain researchers that specialize in food microbiology, concentrating on the separation of some isolated pathogenic microorganisms (STEC) from the infected vegetable or fruit is crucial. The nanotechnology strategy is a key instrument for creating a good and superior solution for the issue at hand using green Ag NPs that were synthesized. Our project is finished by applying the synthesized Ag NPs through the in vivo purpose after dispersing the tested vegetable/fruit in a solution containing Ag NPs and some polymers to format a thin film around the tested vegetable/fruit in an exact concentration lower than the toxicity level of Ag NPs towards invading the STEC and improving the food quality.

The advantages of using the biogenic Ag NPs in the treatment of vegetable/fruit is the cost-effective, eco-friendly, reliable, simplicity of processing, nontoxic at acceptable level, high availability, low energy, low pressure and temperature, no toxic solvents, economic, and ease of technology transfer. Some Ag NPs have positive effects such as improving plant growth and increasing crop production when proper concentrations are used. However, more adverse effects of high Ag NPs concentration have been reported including the inhibition of seed germination, the reduction of photosynthesis and disruption in plant root. According to European Food Safety Authority (EFSA), a food product must not contain more than 50 µg per gram of the item which checked in our work and not exceeds that concentration.

With promising results, natural products have been used to evaluate novel alternative therapy options for STEC infections [54]. The objective of the present study was to evaluate the anti-STEC activity of silver nanoparticles (Ag NPs), which have been shown to possess excellent antibacterial activity against several pathogenic microorganisms [55]. Previous research has demonstrated the importance of Ag NPs because of their unique size, structure, and reactivity. Consequently, it was utilized in numerous applications, such as antibacterial, bio-sensing, catheters, food packaging, textiles, agricultural, anticancer, antioxidant, and drug delivery ([56, 57] and [58]). The antibacterial activity of Ag NPs may be attributed to contacting of silver nanoparticles with bacteria, producing free radicals that cause damage to the cell membrane and pore formation [59]. The difference in the inhibitory percentage of biofilm can be explained by a variety of factors, including the antimicrobial efficacy of the synthesized Ag NPs-clove extract, physical characteristics such as the size of the Ag NPs-clove extract (13 nm), the entrance facilities, and other chemical properties related to the interaction and relationship of the Ag NPs-clove extract with the microbial biofilms [34]. Bacterial adherence to any surface is the first step in biofilm development, it was reported by [60] that Ag NPs attached to the cell surface can block this process by neutralizing the adhesive substances involved in biofilm formation and thus inhibit the biofilm formation process. The expression of polysaccharide by STEC play a major role in attachment [61]. In addition, expression of cellulose was noticed in *E. coli* isolated human gastrointestinal tract suggesting the role of cellulose in attachment and colonization [62].

**Fig. 11:**
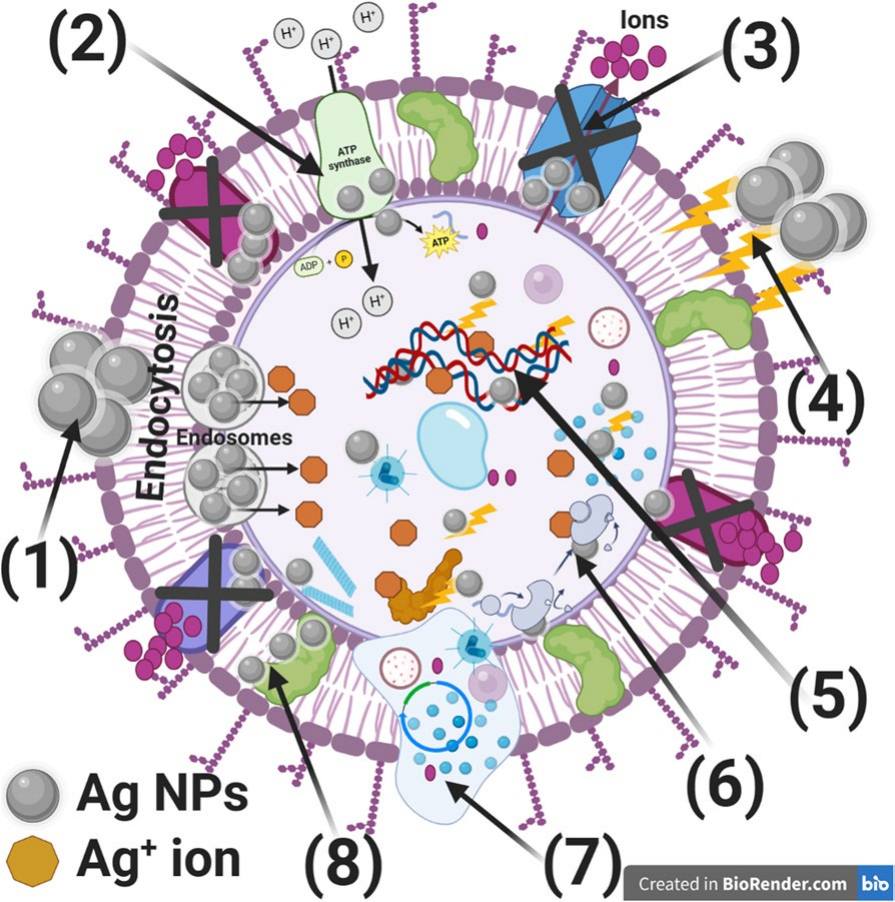
Illustrates the expected reaction mechanisms of the biogenically synthesized Ag NPs against STEC, where 1)—Ag NPs adhere to the *E*. *coli*'s surface and result in membrane breakdown, endocytosis, the creation of endosomes, and altered transport potential, 2) – Ag NPs damages the electron transport chain, 3) – Ag NPs prevent ions from entering and leaving the bacterial cell, 4) – Ag NPs produce and increase ROS, which suggests that the bacterial cell wall is weakening, 5) – Ag NPs enter bacterial cells and interact with cellular organelles (such as DNA), affecting the function of the corresponding cellular components and triggering cell lysis, 6) – Ag NPs interact with enzymes and metabolism, 7) – Ag NPs disrupt the cell membrane and resulting in the leakage of the internal organelles, and 8) – Ag NPs inhibit the membrane protein Additionally, Ag NPs could act as a transporter to effectively release Ag^+^ ions into the cytoplasm and layer, where the presence of proton motive force would cause the pH to drop below 3.0 and encourage the release of Ag^+^ ions, The figure created by BioRender.com

Based on the obtained antibiofilm and antibacterial activity results of Ag NPs, we continued to investigate the mechanism of action of Ag NPs on STEC biofilm producers. Uncertain is the precise mechanism by which Ag NPs affect cells. Figure 11 illustrates the different modes of action of Ag NPs, which include an attachment to the surface of the bacterial cell wall and membrane, penetration into the cell and disruption of intracellular organelles and biomolecules, development of oxidative stress, and modification of signal transduction pathways [63], Fig. 11 showed the different modes of actions of Ag NPs.

The most attachment and accumulation of Ag NPs on the cell surface was observed in Gram-negative bacteria. Ag NPs can enter Gram-negative bacterial cells via porins, which are water-filled channels in the outer membrane. Porins play a crucial role in the passive transmembrane transport of various sizes and charges of hydrophilic molecules. Because the thicker cell wall of Gram-positive bacteria permits silver ions to enter the cytoplasm, Ag NPs exert a greater effect on Gram-negative bacteria than Gram-positive bacteria [64].

The bactericidal properties of Ag NPs are attributed to their ability to interfere with bacterial cell integrity by binding to essential cellular structural components, primarily their SH groups [65]. In addition, they generate reactive oxygen species and free radicals that damage cell walls and inhibit respiratory enzymes [66]. The antibacterial effects of Ag NPs are attributed to the production of free radicals that damage membranes. Ag ions interact strongly with thiol groups in enzymes and phosphorus-containing bases, and Ag NPs may inhibit bacterial cell division and replication, resulting in cell death [67].

## Conclusion

This study revealed that 25 out of 120 samples of fresh vegetables were positive for *E. coli* presence, six isolates were positive for partial (α) blood hemolysis, and three isolates were positive for the presence of Shiga toxin-producing *E. coli* (STEC), two Stx_1_ and one Stx_2_ gene variants. *Syzygium aromaticum* is an effective bioactive compound for fabricating Ag NPs in the presence of varying doses of gamma radiation. It was discovered that increasing the gamma irradiation dose beyond 5.0 kGy increased the size distribution and mean particle size of the produced Ag NPs. The results demonstrated that the product amount and size of the generated Ag NPs are adequately regulated by gamma irradiation. The amount of Ag NPs is greatest with a gamma dosage of 5 kGy with an absorption band at 430 nm. The spherical shapes with mono-dispersed NPs of the biogenic Ag NPs with an average size ranging from 9.18 to 12.98 nm and a mean diameter of 10.99 nm were visible in the SEM and HRTEM images. In the present study it was noticed that Ag NPs was highly effective against the STEC isolates specially the highly drug resistance isolate STX_2_EC (19 ± 2.3 mm) inhibition zone and (11 ± 1.73 and 10 ± 1.73 mm) inhibition zone for STX_1_EC_1_ and STX_1_EC_2_, respectively. The synthesized Ag NPs inhibit the growth and formation of biofilm by the isolated STEC. Therefore, the current study paves the way for a new approach for synthesizing Ag NPs using natural products, and these NPs can be used in various biomedical applications especially against some pathogenic and food-born microbes.

### Supplementary Information

**Additional file 1.**

## Data Availability

The datasets used and/or analyzed during the current study are available from the corresponding author on reasonable request.

## Abbreviations

STEC: Shiga toxin-producing E. coli
Ag NPs: Silver nanoparticles
PCR: Polymerase chain reaction
SEM: Scanning electron microscope
DLS: Dynamic light scattering
HR-TEM: High resolution transmission electron microscope
XRD: X-Ray diffraction
EDX: Energy dispersive X-Ray
FTIR: Fourier transform infrared
MICs: Minimum inhibitory concentrations
HUS: Hemolytic uremic syndrome
EMB: Eosin methylene blue
SMAC: MacConkey agar with sorbitol
PBS: Phosphate buffered saline
CFU: Colony-forming unit
FWHM: Full width at half maxima
O.D.: Optical density
APC: Aerobic plate count
FCC: Face centered cubic
JCPDS: Joint committee on powder diffraction standards
