# Supplementary material for: Detection of hemolytic Shiga toxin-producing Escherichia coli in fresh vegetables and efficiency of phytogenically synthesized silver nanoparticles by Syzygium aromaticum extract and gamma radiation against isolated pathogens
Source: BMC Microbiol. 2023 Sep 18;23:262. doi: 10.1186/s12866-023-02994-8 (PMC10508014; doi:10.1186/s12866-023-02994-8)
Supplement: Supplementary file 1 — Additional file 1. [file 12866_2023_2994_MOESM1_ESM.docx]

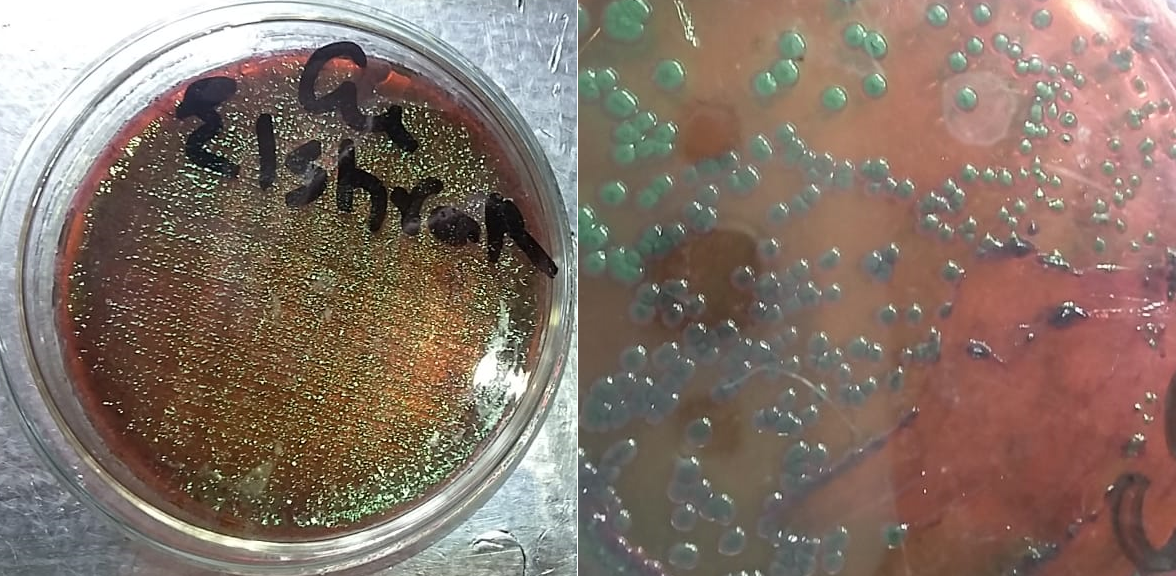


Figure (S1): Isolation of *E. coli* on EMB; colonies with a greenish metallic sheen and black cores.


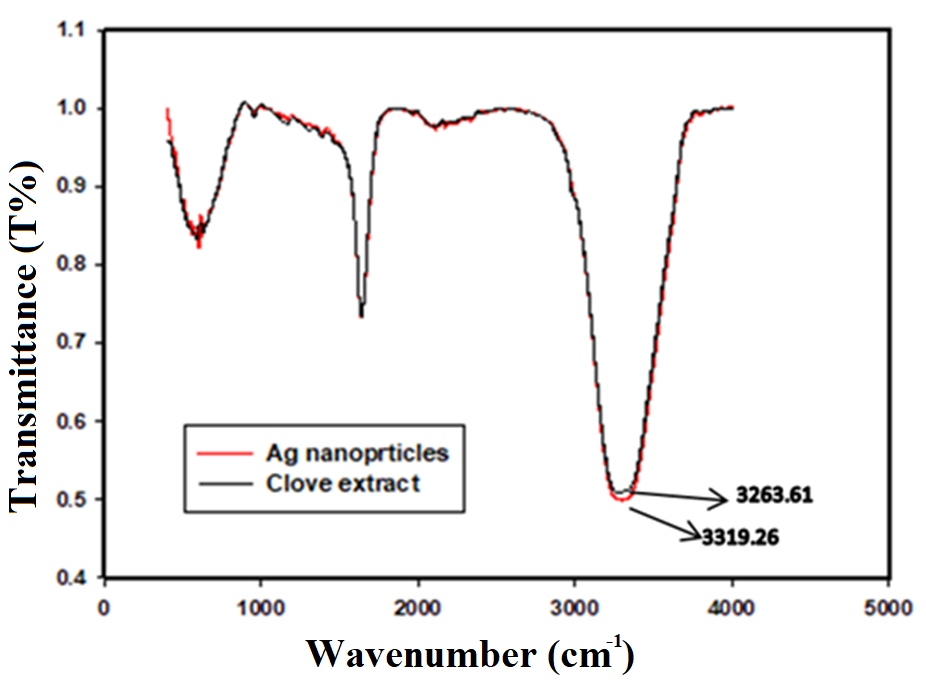


**Figure (S2): Fourier transformed infrared spectroscopy spectrum of biosynthesized Ag NPs.**
